# Virtual reconstruction of the *Canis arnensis* type (Canidae, Mammalia) from the Upper Valdarno Basin (Italy, Early Pleistocene)

**DOI:** 10.1038/s41598-024-53073-5

**Published:** 2024-04-09

**Authors:** S. Bartolini-Lucenti, O. Cirilli, M. Melchionna, P. Raia, Z. J. Tseng, J. J. Flynn, L. Rook

**Affiliations:** 1 Earth Science Department, Paleo[Fab]Lab, University of Florence, via La Pira 4, 50121 Florence, Italy; 2 Institut Català de Paleontologia Miquel Crusafont, Universitat Autònoma de Barcelona, Edifici ICTA-ICP, c/Columnes s/n, Campus de la UAB, 08193 Cerdanyola del Vallès, Spain; 3 Laboratory of Evolutionary Biology, Department of Anatomy, College of Medicine, 520 W St. N.W., Washington, DC 20059 USA; 4 Dipartimento di Scienze della Terra, dell’Ambiente e delle Risorse, Università degli Studi di Napoli Federico II, Via Cinthia 21, 80126 Naples, Italy; 5 Department of Integrative Biology and Museum of Paleontology, University of California, Berkeley, CA 94720 USA; 6 Division of Paleontology, American Museum of Natural History, 200 Central Park West, New York, NY 10024 USA; 7 Changes Foundation, Sapienza Università di Roma, Piazzale Aldo Moro 5, 00185 Rome, Italy

**Keywords:** Palaeontology, Computational biology and bioinformatics, Classification and taxonomy

## Abstract

Taphonomic deformation, whether it be brittle or plastic, is possibly the most influential process hindering the correct understanding of fossil species morphology. This is especially true if the deformation affects type specimens or applies to or obscures taxonomically diagnostic or functionally significant traits. Target Deformation, a recently developed virtual manipulation protocol, was implemented to address this issue by applying landmark-guided restoration of the original, deformed fossils, using undeformed specimens (or parts thereof) of the same species as a reference. The enigmatic Early Pleistocene canid *Canis arnensis* provides a typical example of a fossil species in dire need of virtual restoration. Its lectotype specimen is heavily deformed and none of the few known skulls are well preserved, obscuring the recognition of its systematic and phylogenetic position. Our results indicate that the algorithm effectively countered the lectotype skull’s laterolateral compression and its concomitant rostrocaudal elongation. Morphometrically, comparison of the retrodeformed cranium (IGF 867_W) with other specimens of the same species, and to other fossil and extant canid material, confirms IGF 867_W consistently clusters within *C. arnensis* variability. Overall, the evidence presented here confirms that Target Deformation provides a powerful tool to better characterize complex taxa like *C. arnensis*, whose knowledge is severely affected by the state of preservation of its fossil material.

## Introduction

Canids (Family Canidae) are the most widespread, and one of the most diverse, family of Carnivora^1,2^. In recent years, intense research effort has been dedicated to unravel the taxonomic and phylogenetic affinities between canid species, and within the genus *Canis* in particular^3–9^. Because of its ephemeral and often fragmentary nature, the fossil record fuels this debate by continuously offering new insight on the evolutionary history and phylogenetic polarity of traits^10–13^. The state of preservation of most fossils is often an issue, however, at least when understanding morphological evolution is at stake^14^. Taphonomic deformation (whether brittle, plastic, or a combination of both^14^) alters, or worse, can obscure the geometry of even complete or nearly complete specimens, resulting in the loss of valuable information on the fossil species’ general anatomical shape or proportions and on phylogenetically useful characters^15–18^. This loss of data is especially frustrating when it involves type specimens. Recently, Cirilli et al.^19^ described a new landmark-guided protocol for 3D virtual reconstructions, called Target Deformation (TD), which allows the retroderfomation, i.e. the restoration of the original morphology of plasticly-deformed fossils, using undeformed specimens of the same species as the target. The authors applied the methodology to the holotype of the iconic stenonian horse *Equus stenonis* Cocchi, 1867 (IGF 560), effectively restoring its original and symmetric shape by using partial *E. stenonis* cranial material retrieved from different sites across Europe. Here, we apply TD to perform the virtual reconstruction of yet another renowned Pleistocene species, the Arno River dog *Canis arnensis* Del Campana, 1913. The sample we studied includes two important specimens from the Upper Valdarno Basin (Tuscany, Italy): (i) the lectotype (IGF 867), originally described by Del Campana (1913) and selected by Torre (1967) in his review of Early Pleistocene fossil canids from Tuscany and (ii) a new and better-preserved specimen (IGF 7919V) from the site of Poggio Rosso (Upper Valdarno, Tuscany, Italy) recently described by two of us^20^.

### *Canis arnensis* Del Campana, 1913: an overview

The Arno River dog, *Canis arnensis*, belongs to the large mammal assemblage occupying Western Europe during the Early Pleistocene (ca 2.0–1.6 Ma; biochronologically referred to the late Villafranchian; see Rook and Martínez-Navarro)^20–22^. This timeframe was characterized by the arrival and radiation of members of the genus *Canis* sensu lato into western Eurasia. This massive dispersal event, once known as the “Wolf event”^23^, is nowadays thought to be diachronous and of little biochronological relevance^24^, what remains true is that around 2 Ma, western European *Canis* diversity increased considerably^22^. *Canis arnensis* is a representative of these diverse canid forms^20^. Despite the early description and abundant material, *C. arnensis* is little known in terms of paleobiogeography and evolutionary history of this fossil species. The first insights on this taxon were provided by Torre^25^, with the morphologies and proportions of the lower dentition leading Torre to point out the similarity to a jackal-morphotype as opposed to the coeval Etruscan wolf, *Canis etruscus* and the extant gray wolf, *Canis lupus*. To the contrary, Kurtén^26^ included *C. arnensis* in the “coyote-like dogs”, together with the North American *Canis lepophagus* and *Canis latrans*, thereby excluding a relationship with jackals. In their phylogenetic analysis, Tedford et al.^27^ suggested *C. arnensis* is positioned at the base of a clade of other fossil species of *Canis*, *Xenocyon*, *Cuon* and *Lycaon*. Recent analyses still have not resolved this issue^10^. Part of this longstanding taxonomic and phylogenetic confusion is certainly due to the condition of the type specimens, whose morphologies are obscured by the degree of plastic deformation. Herein, we address this issue, restoring the original shape of the skull by Target Deformation.

### *Canis arnensis* specimens

#### Lectotype—IGF 867, Il Tasso (Upper Valdarno)

**Figure 1:**
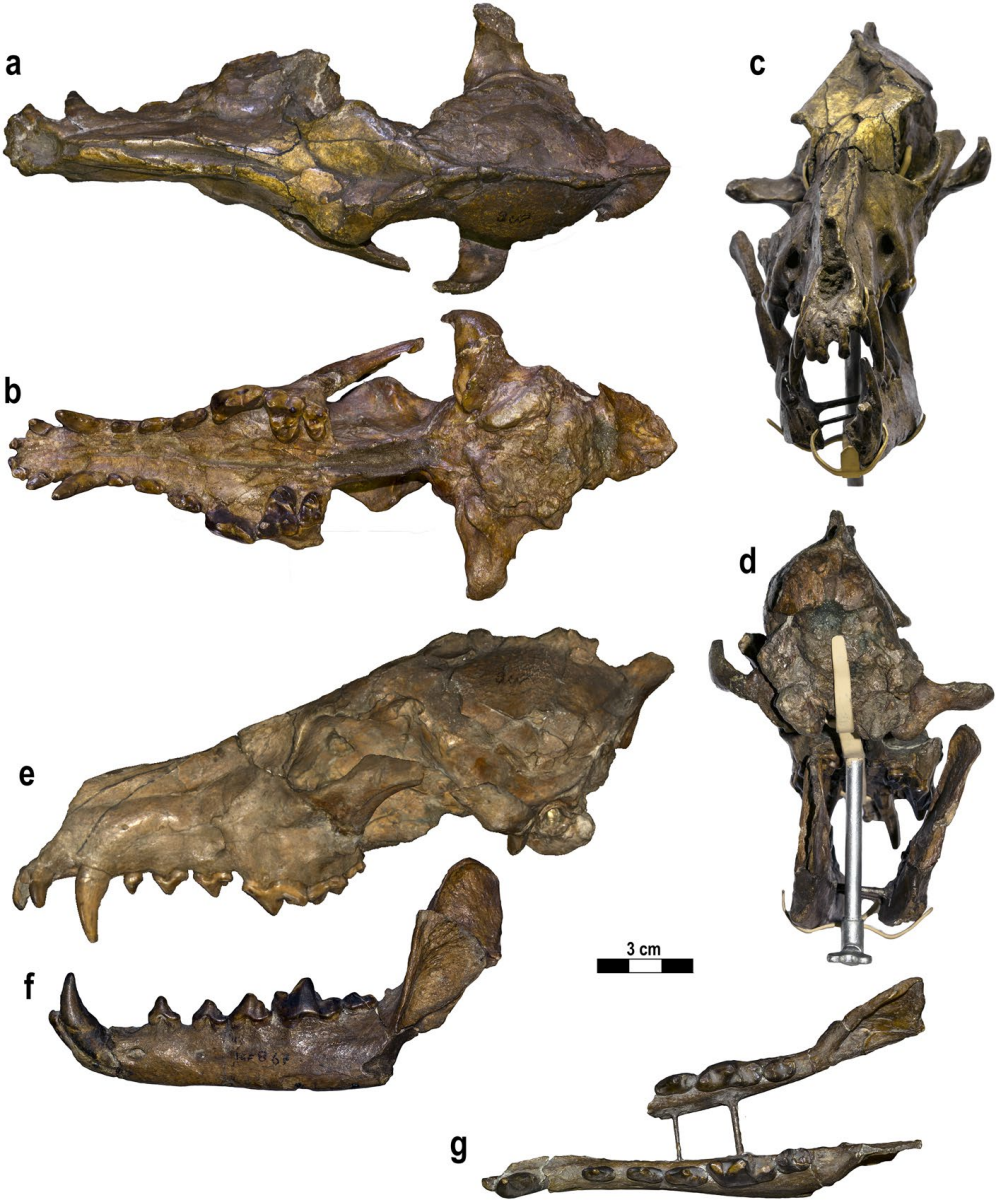
*Canis arnensis* Del Campana, 1913 lectotype (selected by^25^), IGF 867, cranium with partial mandibles from Il Tasso (Upper Valdarno, Tuscany, ~ 1.8 Ma) shown in dorsal (**a**), ventral (**b**), rostral (**c**), occipital (**d**), left lateral (**e**,**f**) and occlusal (**g**) views. Photos of the fossil specimens elaborated in Photoshop CC2019 (https://www.adobe.com/). Figure composition made by S. Bartolini-Lucenti in Inkscape ver. 1.2 (https://inkscape.org/).

In his original description, Del Campana^28^ did not indicate a type specimen of this species, despite providing useful description, comparisons, measurements, and pictures of the abundant material retrieved in the Upper Valdarno outcrops. Torre^25^, in his revision of Villafranchian canids from Tuscany, selected the specimen IGF 867 (Fig. 1), a skull (cranium with both mandibles preserved, although the right hemimandible is fragmentary) with complete dentition, as the lectotype of the species. Herein, we give a brief overview of the lectotype specimen describing the major taphonomic deformation it suffered (Fig. 1). The cranium is fairly complete, missing only fragments of the zygomatic arches and part of the right maxillary bone. During the fossilization process, the specimen underwent extensive plastic deformation, resulting in considerable sagittal (lateral) flattening, visible in dorsal and ventral views. Moreover, the deformation produced a significant heightening of the cranium and possible extension of its original rostrocaudal length. The elongation is further exacerbated by the distorted breadth (e.g., affecting the postorbital constriction, width across the zygomatic processes of the frontals, etc.). The lectotype skull elongation exceeds all other specimens of the hypodigm. This plastic deformation is coupled with a secondary pattern of brittle deformation localized on the braincase, mainly on the right side, and on the left frontal. The compression stresses affecting the cranium also had a slight ventral-ward rotational component, as is visible from the shearing area of the right orbit, frontals, and curved nasals, in dorsal and right lateral views. In ventral view, the palate appears highly laterally compressed, and its right side partially overlaps the left. In the same view, the cranium is laterally arched: compared to the rostrocaudal sagittal plane, the palate and the sphenoid region are tilted relative to one another, with the latter displaced to the left. This is evident especially caudally to the M2. Unlike the cranium, the upper teeth and the mandible fragments were not affected by the deformation.

#### IGF 7919V from Poggio Rosso (Upper Valdarno)

**Figure 2:**
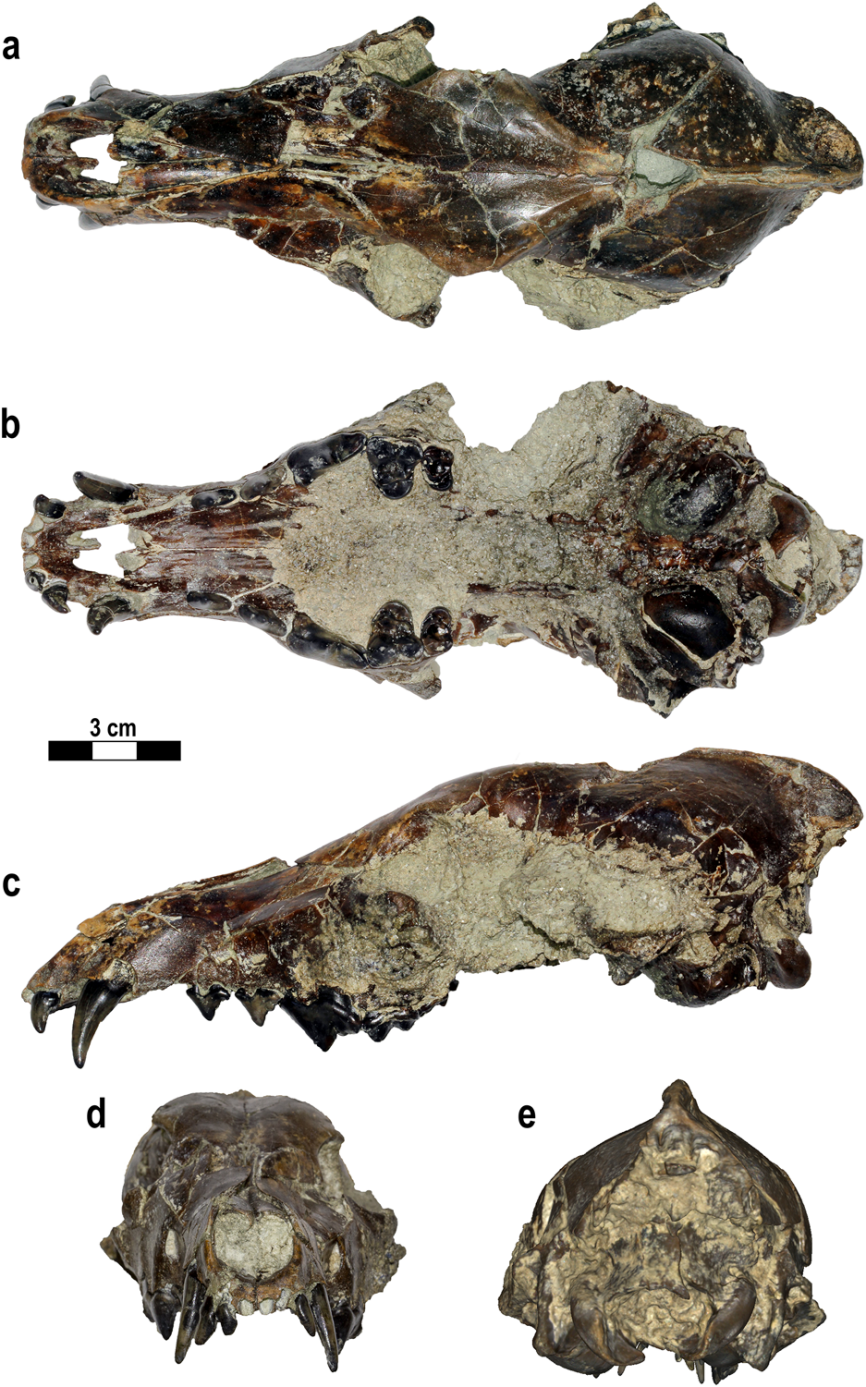
Target specimen, IGF 7919V, cranium with partial mandibles from Poggio Rosso (Faella, Upper Valdarno, Tuscany; ~ 1.9 Ma) selected for retrodeformation of the lectotype of Fig. 1. The specimen is shown in dorsal (**a**), ventral (**b**), left lateral (**c**), rostral (**d**) and occipital (**e**) views. Photos of the fossil specimens elaborated in Photoshop CC2019 (https://www.adobe.com/). Figure composition made by S. Bartolini-Lucenti in Inkscape ver. 1.2 (https://inkscape.org/).

IGF 7919V (Fig. 2) is an almost complete cranium, lacking only the zygomatic arches and the tip of the right zygomatic process of the frontal. In dorsal view, the cranium is modestly elongated and appears rather short. The inion overhangs the condyles, greatly extending beyond them caudally. The nasals are short, reaching the same level of the maxillofrontal suture. The preservation of the tympanic area allows characterization of the auditory region with its large, oval-shaped, and inflated bullae. In ventral view, the medial walls of the bullae are straight and mutually parallel along the midline basicranial axis. The palate is little deformed, showing only a modest rostrocaudal elongation but relatively large width, especially at the level of the canines. IGF 7919V shows some small deformation fractures on the muzzle and braincase. On the dorsal side of the latter, there is a three-sided hole. The maxillary bones and the area rostral to the orbits are damaged, especially on the left side. The right maxilla is broken and overlaps the nasals. The zygomatic processes of the frontals are enlarged and rounded, whereas the postorbital constriction is fairly wide. In rostral view, the cranium shows a slight dorsoventral distortion, resulting in the left side being faintly lower than the right one. Despite these features, IGF 7919V remains the best-preserved cranium of *Canis arnensis*.

## Results

### IGF 7919V retrodeformation and IGF 867 target deformation

**Figure 3:**
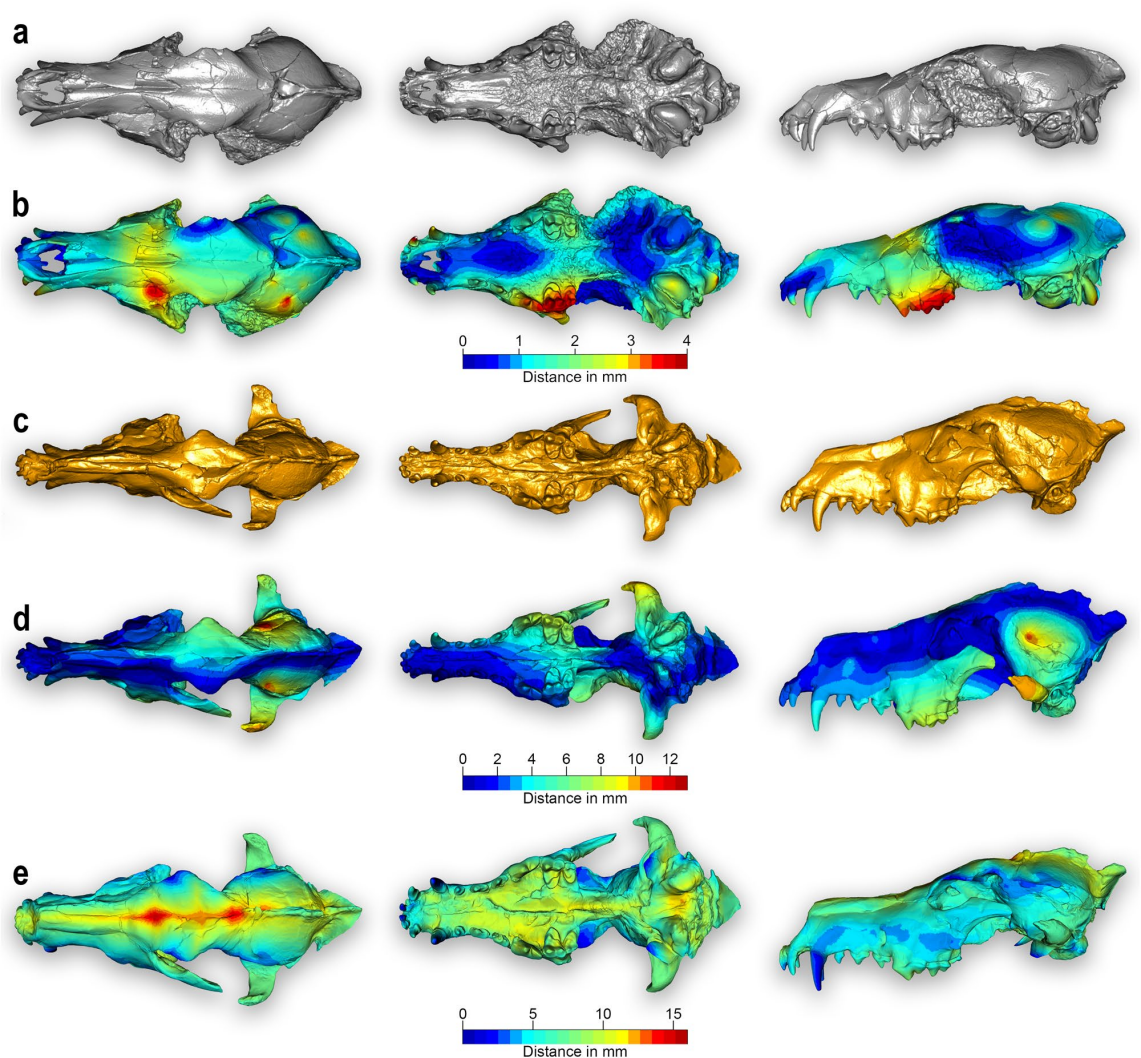
Comparison of the 3D models obtained with Target Deformation. (**a**) IGF 7919V from Poggio Rosso^20^; specimen used as target. (**b**) Result of symmetrization process applied to the target before its use in the Target Deformation protocol. (**c**) IGF 867, type of cranium of *Canis arnensis* and lectotype of the species. (**d**) IGF 867_R from the preliminary symmetrization of the lectotype. (**e**) IGF 867_W, the final target deformation model reconstruction. Color gradient (from blue to red) indicates the increase in mesh vertex displacement in millimeters in IGF 7919V_R (B), IGF 867_R (D) and IGF 867_W (E). Specimens not to scale. Figure composition made by O. Cirilli and S. Bartolini-Lucenti in Photoshop CC2019 (https://www.adobe.com/).

The results of the Retrodeformation and Target Deformation (TD) of the *C. arnensis* crania are shown in Fig. 3. Figure 3a,b represent the target IGF 7919V, as naturally preserved, and the symmetrized target IGF 7919V_R. This model differs from the original specimen for the symmetrization applied via bilateral landmark on IGF 7919V (see “Materials and methods”): the color scale bar in between Fig. 3a,b is obtained from *meshDist()* and is used to evaluate the morphological distance in mm between these two 3D models. Compared to IGF 7919V, IGF 7919V_R is completely symmetrized. The deformed areas present on the frontal dorsal-left side of the cranium of IGF 7919V have been aligned with the right one, as also done for the right parietal bone, resulting in a completely symmetric shape of the braincase in IGF 7919V_R. Moreover, the use of *retroDeformMesh()* permits the restoration of the original morphology of the P4, M1 and M2 by removing their plastic deformation, and the right tympanic bulla has been aligned with the left one. IGF 7919V_R shows an overall typical deformation degree of between 1–2 mm, with the highest degree up to 4 mm due to the good preservation of IGF 7919V (Fig. 3b).

Figure 3c–e compare the results of the original lectotype IGF 867, symmetrization of the lectotype IGF 867_R, and the final model obtained by applying TD to IGF 867 (IGF 867_W). Figure 3c,d show IGF 867 and IGF 867_R in dorsal, ventral and lateral views, with a color scale bar obtained from *meshDist(),* to evaluate the morphological distance in mm between the two 3D models. The retrodeformation performed on IGF 867 yields the fully symmetrical IGF 867_R. Compared to IGF 867, IGF 867_R displays a maximal deformation of 12 mm between the parietal bones (both left and right side). Moreover, the retrodeformation removed the slight ventral rotational component which affects the left side of the cranium, resulting in a fully symmetric morphology of the palate and frontal bones (green areas in Fig. 3d). In ventral view, IGF 867_R renders the two upper teeth rows completely parallel. The palatine processes of the muzzle remain mediolaterally compressed, but its right side no longer overlaps the left one, and the sphenoid region is now aligned.

Figure 3e shows IGF 867_W, the 3D model generated by applying the TD, with distances calculated from IGF 876_R. The TD has completely removed the laterolateral compression still present after initial retrodeformation in IGF 867_R. The general morphology is comparable with the target specimen IGF 7919V_R, showing deformation on the nasals, maxilla, frontal bone, postorbital process, and parietal bones. In dorsal view, the highest degree of deformation is reached in the postorbital constriction and in parietal bones (15 mm), whereas the zygomatic arches and nasals have been retrodeformed without a landmark configuration, due the absence of homologous landmarks in IGF 7919V_R. In ventral view, the premaxilla, maxilla, palatine process, and the basisphenoid and basioccipital areas are improved with a deformation between 5 and 10 mm, resulting in a very similar morphology to the target of IGF 7919V_R. TD has further reduced the rostro-caudal length and height of the cranium (lateral view), the two principal taphonomic deformations affecting both IGF 867 and IGF 867_R, resulting in a shorter, higher, and larger cranium. Overall, no relevant blue areas (regions of high deformation) are visible in IGF 867_W, demonstrating that latero-lateral compression affected the whole surface of the cranium, with the exception of the upper teeth. In lateral view, the absence of homologous anatomical points between IGF 867_R and IGF 7919V_R on the nasals and on the dorsal surface of the maxilla led to a more pronounced deflection of the IGF 867_W latter morphology. Comparative measurements of the original lectotype IGF 867, retrodeformed IGF 867_R, and TD reconstructed IGF 867_W are reported in Table 1.

**Table 1:** List of cranial measurements of the *Canis arnensis* lectotype IGF 867, the first step 3D model IGF 867_R, and the final Target Deformation model IGF 867_W.

	IGF 867	IGF 867_R	IGF 867_W
TL	203.9	201.4	186.4
NCL	94.6	93.8	91.9
FL	114.5	114.2	99.2
SCL	98.7	96.9	84.8
GNL	73.1	69.6	66.3
Eu	41.7	49.4	56.1
Ect	42.6	45.4	45.8
PoCW	24.4	24.7	36.0
IoD	27.6	26.3	35.1
GHOr	21.4	28.6	19.8
SH	60	51.2	51.0
AB	41.6	44.1	45.0
GWOT	49.4	54.3	59.4
PL	97.6	93.1	94.5
ECW	21.8	21.7	34.6
GPW	43.2	47.4	53.1
HPW	19.1	18.8	28.4
MOH	30.3	31.5	26.5
PWP1	12.5	12.2	16.4
PWP2	14.8	15	19.7
M2B	46.1	46.2	45.5

All measurements in mm. Measurement abbreviations: AB, height of the cranium without the sagittal crest (inion-basion); Ect, width across the zygomatic processes of the frontals; ECW, width of the muzzle at level of the upper canine; Eu, greatest neurocranium width (eurion-eurion); FL, facial length; GHOr, greatest height of the orbit; GPW, greatest palatal width; GWOT, greatest width of the occipital triangle; hemipalate width; IoD, interorbital distance, minimum width of the glabella; M2B, length of the cranium between the M2 and the tympanic bulla (measured between the distal side of the M2 and the rostral margin of the bulla); MOH, maxilla-orbit height (from M1 alveolus to the orbit); NCL, neurocranial length; PL, palatal length (staphion-prosthion); PoCW, smallest width of postorbital constriction; PWP1, palatal width at the level of the P1 (measured between the lingual side of the P1 alveolus); PWP2, palatal width at the level of the P2 (measured between the lingual side of the P2 alveolus); SCL, splanchnocranial length (nasion-prosthion); SH, skull height (with sagittal crest); TL, total length of the cranium (inion-prosthion).

### Morphometric analyses

**Figure 4:**
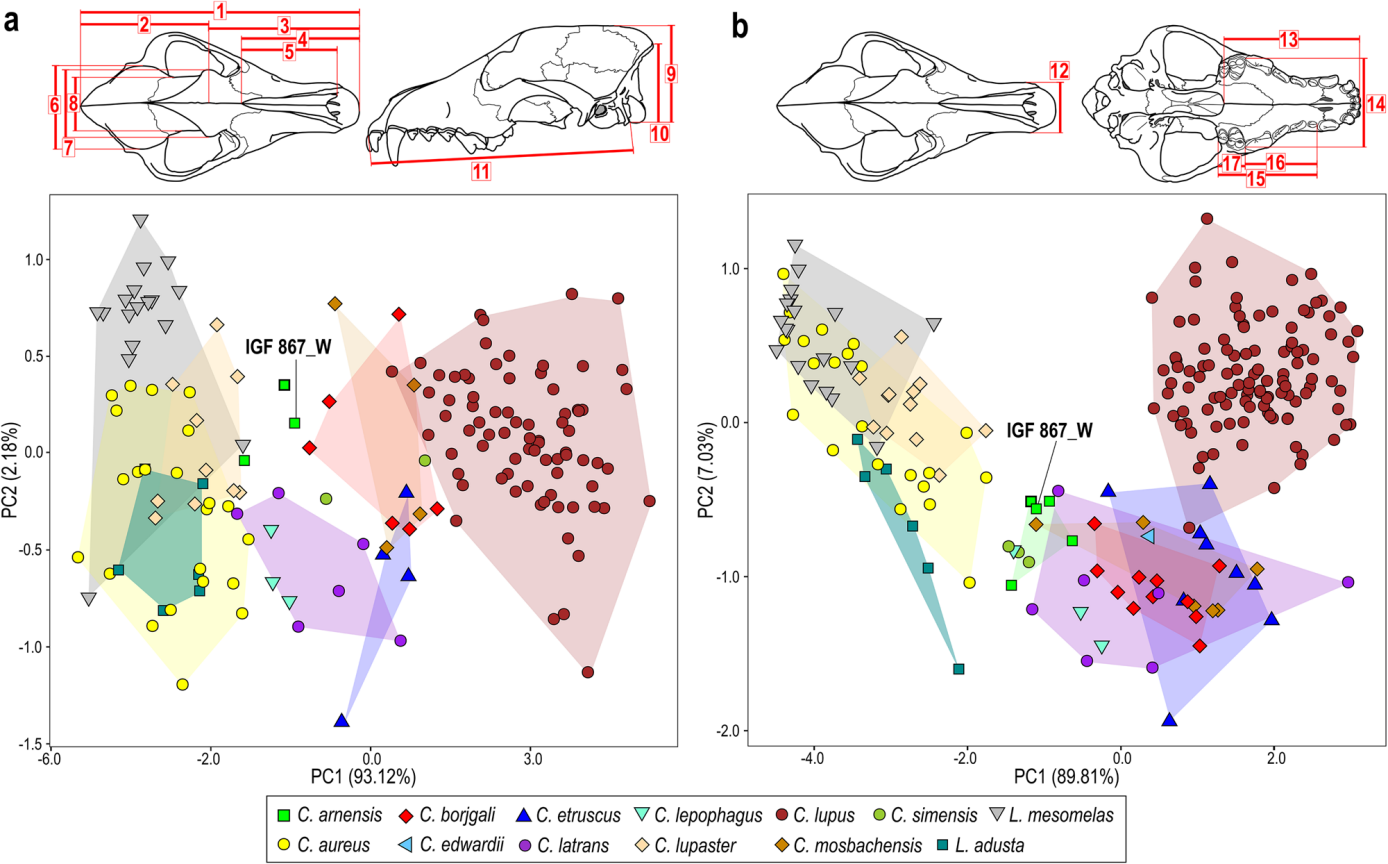
Principal component analyses (PCA) on selected craniodental measurements comparing IGF 867_W with a set of extant and fossil species from North America and Eurasia of the genera *Canis* and *Lupulella* and other specimens ascribed to *Canis arnensis* (the bold green square represents the target specimen IGF 7919V). (**a**) PCA on eleven cranial variables and (**b**) PCA on six craniodental variables, as shown in the line drawings above the plots (see list below). Numeric code for measurements used: 1, total length of the cranium (inion-prosthion; acronym: TL); 2, neurocranial length (NCL); 3, facial length (FL); 4, splanchnocranial length (nasion-prosthion; acronym: SCL); 5, greatest length of the nasals (GNL); 6, greatest neurocranium width (eurion-eurion; acronym: Eu); 7, width across the zygomatic processes of the frontals (Ect); 8, smallest width of postorbital constriction (PoCW); 9, skull height with sagittal crest (SH); 10, height of the cranium without the sagittal crest (inion-basion; acronym: AB); 11, basal length of the cranium (basion-prosthion; acronym: BL); 12, width of the muzzle at level of the upper canine (ECW); 13, rostrocaudal length of the palate (staphion-prosthion; acronym: PL); 14, greatest palatal width (GPW); 15, length of the upper toothrow, between the mesial side of the P1 and the distal one of the M2 (P1-M2 L); 16, length of the premolar row, between the mesial side of the P1 and the distal one of the P4 (P1-P4 L); 17, length of the upper molar row, between the mesial side of the M1 and the distal one of the M2 (M1-M2 L). Graphs obtained using the R function *ggplot()* (package ‘*ggplot2*’ v.3.4.0^58^). Line drawing of canid skull and figure composition made by S. Bartolini-Lucenti in Inkscape ver. 1.2 (https://inkscape.org/).

The PCA used to evaluate the performance of the Retrodeformation and TD of IGF 867 is reported in Fig. 4. We chose the most distinctive cranial and dental measurements to assess the position of IGF 867_W in the morphospace of cranial variability for this canid species (Fig. 4a,b). Loadings and parameters are reported in the Supplementary Table S1. Figure 4a shows the component analysis performed on eleven cranial variables. PC1 accounts for 93.12% of the variance and has positive and similar loadings for all the analyzed variables (Supplementary Table S1). Along the PC1 axis, the extant taxa are fairly well separated into three clusters (Fig. 4a). On the negative side of this axis, a large group includes African and Eurasian jackal-like taxa (e.g., *C. aureus*, *C. lupaster*, *L. adusta* and *L. mesomelas*). *Canis lupus* is situated on the opposite side, with positive values; and *Canis latrans* and *C. simensis* lie in between these. Fossil forms are placed close to this latter, intermediate group. *Canis lepophagus* displays negative PC1 values and its variability overlaps with that of *C. latrans*. In contrast, *C. etruscus*, *C. mosbachensis* and *C. borjgali* range across low negative and positive values of PC1, and are situated closest to *C. lupus*. Along PC1, the three specimens of *C. arnensis* lie close to *C. lepophagus*. PC2 only accounts for 2.18% of total variance. It is dominated positively by cranium width and height (the latter including the sagittal crest), and negatively by skull length, especially by the greatest length of the nasals. All the investigated taxa overlap to a large extent on PC2 (Fig. 4a). Along this axis, specimens of *C. arnensis* are only slightly separated from each other. The retrodeformed type specimen (IGF 867_W) falls in between the other undeformed *C. arnensis* specimens used as comparative material (i.e., IGF 869; IGF 7919V, the target specimen) (Fig. 4a).

A second principal component analysis was performed on six craniodental variables (as shown on the top of Fig. 4b). In this analysis, PC1 accounts for 89.84% of the total variance. As in the previous PC analysis, all the variables have comparable loadings (see Supplementary Table S2). Again, along this axis extant canids are arranged in three clusters: jackal-like canids on the negative part of PC1, *C. lupus* on its positive end, and *C. latrans* and *C. simensis* in between (Fig. 4b). The position of fossil species is consistent with the result of Fig. 4a: *C. borjgali*, *C. etruscus* and *C. mosbachensis* with comparable values and closer to *C. lupus* variability; with *C. arnensis* located closer to extant jackal-like canids. PC2 explains ~ 7% of the variance, and is positively influenced by the length of the palate and palate width measured at the level of the canine and at its maximum point (i.e., at the P4-M1 junction). In contrast, toothrow length, especially the molar row, has a negative impact on this axis. Along the PC2 axis the majority of the extant sample has positive values, with the exception of *C. latrans*, *C. simensis*, *L. adusta*, and some *C. aureus*. The fossil species are all located in the negative part of the PC2 axis (Fig. 4b). In this second PCA, the model obtained with the TD protocol, IGF 867_W, is located entirely within the convex hull of *C. arnensis*.

Overall, as visible in Fig. 4, IGF 867_W is positioned within the range of variability of extant and fossil canids, and particularly is always close to other specimens of *C. arnensis*. These results confirm the quality of the protocol applied. The 3D model IGF 867_W can be considered as coherent with the skull morphology of fossil *Canis* s.l. species.

**Figure 5:**
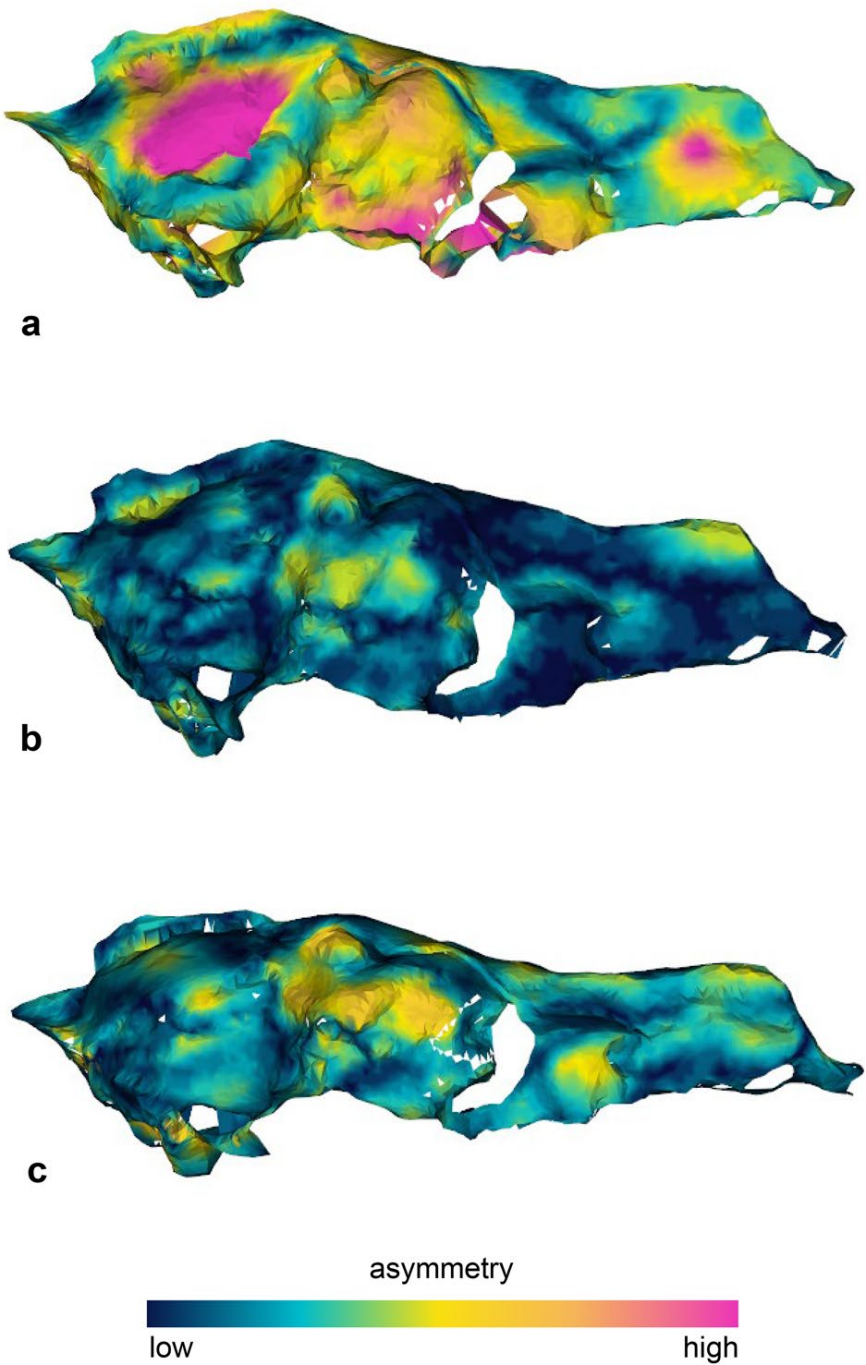
Visualization of the degree of bilateral asymmetry in the original skull of the *Canis arnensis* lectotype IGF 867 (**a**), the symmetry retrodeformed model IGF 867_R (**b**), and the final target deformation reconstruction IGF 867_W (**c**). Figure composition made by M. Melchionna in Photoshop CC2019 (https://www.adobe.com/).

The application of *show.asymmetry* methodology^29^ reveals and maps the extensive deformation affecting IGF 867 (Fig. 5a). As expected, the retrodeformation protocol almost entirely removes the asymmetry around the midline (Fig. 5b). Application of TD comes at the cost of reintroducing some minor asymmetry (Fig. 5c), but provides a biologically sounder shape that is closer to the less deformed target (IGF 7919V, see Fig. 3).

## Discussion and conclusion

The enhanced retrodeformation performed by implementing the TDmethodology^19^ on the lectotype of *C. arnensis* IGF 867 (Fig. 1), using the cranium from Poggio Rosso IGF 7919V (Fig. 2) as a target reference specimen, produced encouraging results. The choice of IGF 7919V as a target to guide the retrodeformation is necessary because of the absence of undeformed or non-damaged specimens of *C. arnensis* (see the record reported by previous authors^20,25,28^), as none other complete or partial cranial fragments of this species have yet been recovered. This paucity of material did not permit accounting for the fractures and displaced right side of the nasomaxillary region of IGF 7919V, as is visible in the final TD model IGF 867_W (Fig. 3). Nevertheless, this 3D TD model (IGF 867_W) has produced a well restored digital specimen of the *C. arnensis* lectotype, IGF 867, more closely representing the in vivo shape and morphology of this individual. The morphometric analyses (Fig. 4) support the validity of the obtained 3D model of the retrodeformed type (IGF 867_W).

The TD reconstruction and its morphometric results helped clarify some of the *known unknowns* (sensu Jackson^30^) on this unusual Early Pleistocene *Canis* species. During the diversification of Canini across Eurasia during the Late Pliocene, *Canis* (sensu lato) dispersed across Eurasia and Africa after 3.5 Ma^24,31^. In western Eurasia the undoubted occurrences of *Canis* spp. are those from the Early Pleistocene of Senèze, France^32^ (2.2–2.1 Ma see^33^), Coste S. Giacomo and localities of its faunal unit, Italy (~ 2.1 Ma^34,35^), and Olivola faunal unit localities (Spain^36^; Italy^20,37^; Greece^38^).Thus, *C. etruscus* and *C. arnensis* represent the two most relevant *Canis* species of the early Early Pleistocene of Europe. In sharp contrast with *C. etruscus*, whose features, taxonomy, and even diet are well-characterized and described^37,39,40^, *C. arnensis* is far less well understood. Part of its enigmatic nature is related to the paucity of remains: most of the specimens come from the Upper Valdarno basin (where the type locality occurs), whereas a few rare reports related medium-sized canids to this form in Italy (Frattaguida^41^), France (the above-mentioned Senèze) and Greece^42^. All those localities are limited to the end of the Gelasian-early Calabrian, ca 2.2–1.6 Ma^21^. If the scantiness of the fossil record is an issue, the state of preservation of the few fossils even more heavily hinders taxonomic, phylogenetic and even paleoecological interpretations. The combined effect of these two biases could explain the poor resolution of the phylogenetic interpretations that considered *C. arnensis* within their ingroup. Torre^25^ was the first to reappraise *C. arnensis* after Del Campana’s^28^ description. Torre’s analyses focused on the comparison between the Arno River dog, *C. etruscus*, and extant Eurasian and African canids. In his interpretation, Torre^25^ concluded that *C. arnensis* more closely resembles Eurasian and African jackals rather than *C. etruscus* or *C. lupus*. In contrast, Kurtén^26^ proposed that *C. arnensis* belonged to a group/lineage of Holarctic coyote-like dogs (i.e., taxa closely allied to *C. latrans*) that characterized North America and Eurasia from the Early Pliocene (with *C. lepophagus*) through the Early Pleistocene (with *C. priscolatrans*—sensu^26^, see below—in North America and *C. arnensis* in Europe) to the extant North American coyotes.

As Kurtén^26^ correctly concluded, the exclusion of *C. latrans* from his analysis led Torre^25^ to overestimate the dental affinity of *C. arnensis* with the extant *C. aureus* (s.l., corresponding today to *C. lupaster* and the Eurasian populations of *C. aureus*), *Lupulella mesomelas,* and *L. adusta*. Indeed, dimensionally (e.g., body size, dentognathic variables) *C. latrans* and *C. arnensis* are much more similar to each other than smaller jackals, as pointed out by Kurtén^26^. Their size similarities are also visible in our Fig. 4. Regarding Kurtén’s^26^ interpretation of *C. arnensis* as conspecific with *C. priscolatrans,* that hypothesis is difficult to assess in full, primarily because *C. priscolatrans* is now regarded as a *nomen dubium*. Tedford et al.^27^ recognized that *C. priscolatrans* sensu Kurtén^26^ is made up of three taxa: *C. edwardii* (as indicated also by Nowak^43^), part of fossil *C. latrans,* and even the hypercarnivorous ‘*C.’ armbrusteri* (as a consequence of the original material used to describe *C. priscolatrans*^44^). On both morphological and phylogenetic grounds we discount the position of ‘*C.*’ *armbrusteri* as allied to its nearly coeval *C. arnensis,* since ‘*C.*’ *armbrusteri* possess hypercarnivorous teeth and dentognathic morphology sharply differing from *C. arnensis*. Moreover, ‘*C.*’ *armbrusteri* is generally viewed as the ancestor of *‘Canis’ dirus* Leidy, 1858 (see Tedford et al.^27^). According to the recent discovery by Perri et al.^13^ that the dire wolf belongs to a separate lineage of North American Canini that differentiated around 5 Ma, and now referred to *Aenocyon dirus*, allying ‘*C.*’ *armbrusteri* to *Aenocyon* seems at least a more plausible option. *Canis edwardii* and *C. latrans* show some interesting patterns in our analyses. The results of the principal component analyses based on cranial variables clearly show that *C. arnensis* differs substantially from *C. edwardii* (Fig. 4b), regardless of whether the original, deformed types or retrodeformed or TD reconstructed specimens are considered. It should be noted that Kurtén^26^ focused on dental variables, which have been regarded as informative primarily about dietary habits^12,39,45^ whereas cranial ones show greater reliability in terms of plausible relationships^46^. Regarding the coyote, although *C. arnensis* lies close to the extant *C. latrans* in the PCA (Fig. 4) and there are superficial resemblances between the crania of these two species, the hypothesis of an affinity between *C. arnensis* and *C. latrans* does not seem to be supported here. Phylogenetic analyses support this distinction, as Tedford et al.^27^ suggested an origin of *C. latrans* from *C. edwardii* rather than *C. arnensis* (which roots at the base of *Canis* s. l. group in their analysis). Similarly, Zrzavý et al.^10^ showed the phylogenetic distance between these latter three taxa. As far as *C. lepophagus* is concerned, Kurtén^26^ considered it anatomically primitive compared to *C. edwardii* and *C. arnensis,* as the first member of the lineage leading to the coyotes. Although largely unresolved when it comes to stem *Canis*, current phylogenetic interpretations agree with the hypothesis of a basal position for *C. lepophagus* in *Canis* clade^10^ without a direct nor a close relationship with *C. arnensis*. Moreover, *C. lepophagus* is the oldest known species of the genus *Canis,* after the recent attribution of ‘*Canis*’ *ferox* Miller and Carranza-Castañeda, 1998 to *Eucyon*^12^. The distinctions of *C. lepophagus* and *C. arnensis* occurrences are not only geographical but also temporal: Tedford et al.^27^ suggested that *C. lepophagus* spans 4.9–1.8 Ma while *C. arnensis* is much younger (ca 2.2–1.6 Ma). Recently, some authors^47^ noted that some North American occurrences referred to *C. lepophagus* should be referred differently (i.e., *Canis* aff. *C. lepophagus*, possibly a new species, and a *Canis* sp. from Hagerman and Rexroad). Nevertheless, even considering these probable attributions, the temporal gap between *C. lepophagus*-like canids and *C. arnensis* remains wide. Despite the superficial resemblance, and comparable metric proximity between *C. lepophagus* and the deformed lectotype of *C. arnensis* IGF 867, the retrodeformed IGF 867_W is always distinct from *C. lepophagus* (Fig. 4). These new TD results favor the interpretation put forward by some of the authors of the present paper^20^ in considering the morphological and morphometric variability of *C. arnensis* as more ‘jackal-like’, awaiting better understanding of the phylogenetic position of this canid.

By applying the TD protocol to the heavily deformed lectotype specimen of *C. arnensis* (IGF 867), we produced a biologically sound reconstruction of the lectotype that is consistent with the proportions of other Canidae, as indicated by the results of the morphometric analyses (Fig. 4). Application of the TD methodology clearly improves the anatomical characterization of important but enigmatic taxa like *C. arnensis*, whose understanding is severely affected by the preservation of available fossils. Applying the TD methodology is urged for other such taxa in the fossil record.

The target deformation reconstruction method applied to the fossil *Canis arnensis* lectotype represents not only an advance in permitting quantitative morphometric analyses of this key specimen, as demonstrated in this study, but also significantly in its future potential to update or extract additional taxonomic characteristics that may have been obscured by the post-mortem deformation of the skull. Specifically, in addition to the morphometric distinctiveness of the TD retrodeformed specimen apparent in the PCA, the phylogenetic uncertainties associated with this taxon in past analyses^10,27^ could be lessened by explicitly coding a phylogenetic matrix or identifying taxonomically diagnostic discrete characters from the TD retrodeformed model of the lectotype. Although beyond the scope of the current study, target deformation provides the capacity for such expanded analyses in the future.

## Materials and methods

### *Canis arnensis* digital models

The specimens of *C. arnensis* here considered are housed in the collections of the Geology and Paleontology Museum of the University of Florence. The 3D digital models used in the analysis, the lectotype IGF 867 and the Poggio Rosso specimen IGF 7919V were acquired using the structural light 3D Scanner Artec Space Spider. The technical specifications of the Artec Space Spider include a 3D point accuracy of up to 0.05 mm, with a 3D resolution of 0.1 mm, a 1.3 megapixel resolution which allows to capture the texture of the object digitalized, a 3D reconstruction rate for real time fusion up to 7.5 frames per second and a data acquisition speed of 1 million points each second of recording (https://www.artec3d.com). The 3D scanner works with blue LED 3D light source. IGF 867 and IGF 7919V were scanned with Artec Space Spider following the methodology of Bartolini Lucenti and Rook^48^: obtaining two partial scans each (one in dorsal and one in ventral), to acquire the external surface geometry. These scans were aligned through definition of homologous morphological landmarks by using the software Artec Studio 17 Professional ver. X64 17.1.2.15, and the final meshes were generated. The 3D models were exported in the .obj and .ply with vertex polygon format.

### Workflow of the 3D virtual reconstructions

**Figure 6:**
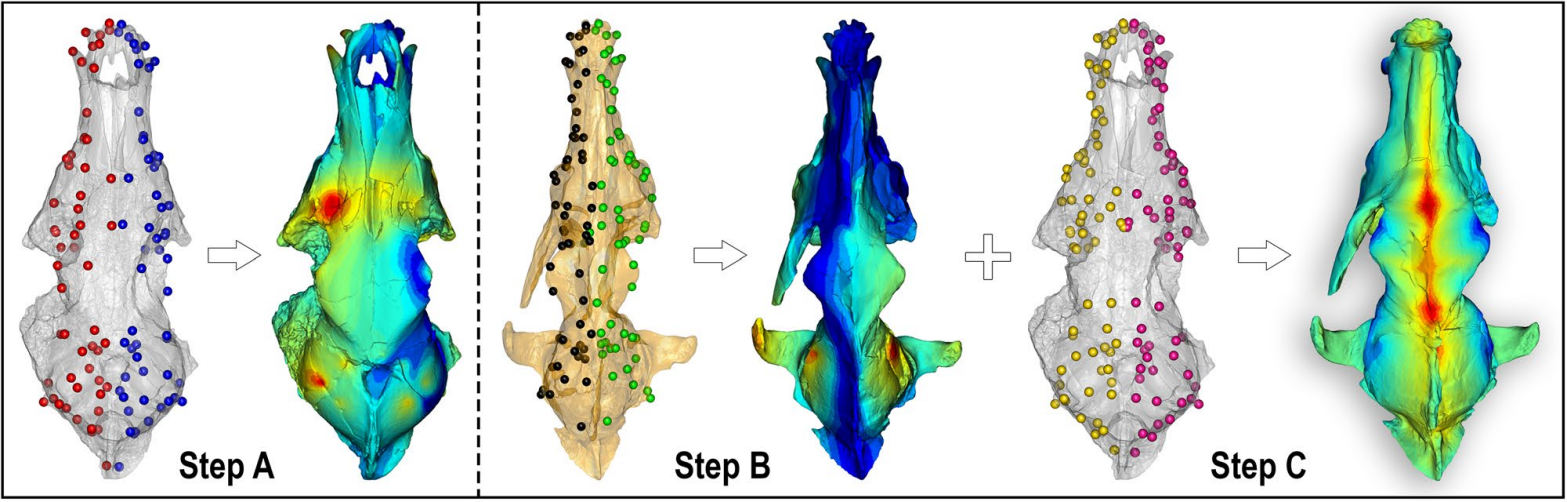
Methodological steps of Target Deformation applied to *C. arnensis*. Step A: symmetrization of IGF 7919V (the selected target) using *retroDeformMesh()* on the basis of 112 bilateral landmarks (red: left side landmarks, blue: right side landmarks), obtaining a corrected model IGF 7919V_R. Step B: same procedure as for IGF 7919V to get symmetrization of IGF 867 based on 156 bilateral landmarks (black: left side landmarks, green: right side landmarks) obtaining an improved model of the lectotype (IGF 867_R). Step C: retrodeformation of IGF 867_R using *tps3d()* on a target set of 152 homologous bilateral landmarks (yellow: left side landmarks, pink: right side landmarks; here shown on IGF 7919V_R) obtaining the final target deformation model, IGF 867_W. Specimens not to scale. Color maps are the same of Fig. 3, explaining the degree of retrodeformation; not in scale with one another. Figure composition made by O. Cirilli and S. Bartolini-Lucenti in Photoshop CC2019 (https://www.adobe.com/).

The procedure developed in this contribution is performed in R Environment v. 4.2.2 (R Core Team, 2022) and it includes a combination of the digital techniques described in recent papers^18,19^. The workflow includes three consecutive steps, as follows (Fig. 6):

1. Perform the retrodeformation of the *C. arnensis* cranium from Poggio Rosso IGF 7919V by using a bilateral landmark configuration to symmetrize the target specimen IGF 7919V_R.
2. Perform the retrodeformation of the *C. arnensis* lectotype, the cranium IGF 867, using a bilateral landmark configuration to restore the symmetry of the lectotype, IGF 867_R.
3. Warp the retrodeformed lectotype onto the retrodeformed IGF 7919V_R to obtain the final TD (Target Deformation) mesh of the *C. arnensis* lectotype, IGF 867_W.

All 3D meshes in this workflow have been standardized to 500.000 triangles to be more versatile in R. Moreover, we sampled only landmarks and not semi-landmarks due to the irregularity of the surfaces of the specimens considered in the analyses, e.g., due to cracks and fragile deformations of the crania. The landmark configurations were manually sampled on Amira software (ver. 5.4.5; www.thermofisher.com/amira-avizo). Landmark positioning protocol follows the requirements of Cirilli et al.^19^.

The two retrodeformations undertaken on the original crania IGF 7919V and IGF 867 were performed with the function *retroDeformMesh()* embedded in the Morpho R package^49^. The *retroDeformMesh()* algorithm was implemented by Schlager et al.^18^ and it computes a non-linear symmetrisation detecting multiple local affine deformations and reducing the interpolation performed by thin-plate spline, TPS^50^. This function uses a set of bilateral landmarks for which their centroid is computed for each pair of landmarks.

We performed retrodeformation of the IGF 7919V 3D mesh, with a set of 112 bilateral landmarks (Fig. 3, Step A) that defines the complete morphology of the cranium. As reported above, the aim of this retrodeformation is to restore the original symmetry of IGF 7919V. This process aims to remove asymmetric alterations due to taphonomic processes which affected the target specimen. At the end of this first process, we obtained IGF 7919V_R, the completely symmetrized 3D mesh of the Poggio Rosso cranium.

With the same procedure, we performed the retrodeformation on IGF 867 with an expanded set of 156 bilateral landmarks, to restore its original symmetry, with the larger set of landmarks possible because of better preservation (Fig. 3, Step B). At the end of this process, we obtained IGF 867_R, the symmetrized 3D mesh of the *C. arnensis* lectotype.

Finally, to undertake the Target Deformation for the lectotype IGF 867, we selected a new landmark configuration of 152 homologous landmarks (Fig. 3, Step C) that could be identified on both IGF 867_R and IGF 7919V_R, and we warped IGF 867_R using the new IGF 7919V_R landmark set. The warping process was performed through *tps3d(),* an R function included in the package Morpho (Schlager, 2017) which deforms a set of coordinates or a mesh via thin plate spline transformation based on a reference and a target configuration. This final step produced the retrodeformed and warped-to-target mesh of the *C. arnensis* lectotype, IGF 867_W.

To compare results between the retrodeformed and warped 3D meshes relative to the original ones, we computed and visualize pairwise surface distances with the function *meshDist* (‘Morpho’ R package by Schlager, 2017) between IGF 7919V and IGF 7919V_R, and IGF 867, IGF 867_R and IGF 867_W. The differences between surfaces were computed as Euclidean distances between corresponding points of the surfaces. Then we applied the *show.asymmetry* algorithm^29^, a landmark-based procedure which allows visualization and measurement of the left–right asymmetry of each specimen; asymmetry equals the square root of the sum of the squared distances between each landmark pairs. By implementing *show.asymmetry*, the asymmetry pattern is automatically visualized on one half of the object surface.

The complete set of data and scripts (including the R script, the original IGF 7919V and IGF 867 3D meshes and the landmark sets) to replicate this workflow is provided in at the following link: 10.5281/zenodo.10418654.

### Morphological and multivariate morphometric comparison

The description focuses on relevant morphological features, following those of Torre^25^ and previous work by some authors of this paper on *C. arnensis*^20^. Teeth nomenclature follows Bartolini-Lucenti & Spassov^51^. To assess the efficacy of the retrodeformed and warped 3D models, we performed a Principal Component Analysis (PCA) on linear measurements the dataset used in Bartolini-Lucenti et al.^40^ and compared IGF 7919V_R and IGF 867_W with a set of fossil and extant canids, inclusive of the original *C. arnensis* lectotype IGF 867. The extant species included here are *Canis aureus*, *Canis latrans*, *Canis lupaster*, *Canis lupus*, *Canis simensis*, *Lupulella adusta*, and *Lupulella mesomelas*. The fossil species considered are: *Canis arnensis* from Upper Valdarno (IGF 869 from Il Tasso; IGF 7919V, IGF 8657V, IGF 8684V from Poggio Rosso); *Canis borjgali* from Dmanisi (D64, D301, D1368, D2314, D3420, D4376, D4510, D5553, D5656, Dm.50/52.3B1.34, unnumbered specimen with provisional number 01/D01); *Canis edwardii* from North America (AMNH F:AM 63100 from Tusker Fauna, 111 Ranch at Dry Mountain locality); *Canis etruscus* from Europe (IGF 4407, IGF 4410 from Olivola; IGF 12867, IGF 14150, MPM 693, MPM 694 from Upper Valdarno; SBAU 337628, SBAU337646 from Pantalla^37^; APL 522 from Apollonia-1^52^); *Canis lepophagus* from North America (AMNH F:AM 63091 from Saint David Quarry, Saint David Formation; AMNH FM 104782—cast of WTUC 760—and AMNH FM 104783—cast of the type WTUC 881—both from Cita Canyon, Randall County^27^); *Canis mosbachensis* from Europe (IMEDEA-C1, IMEDEA-C2 from Cueva Victoria; DE not numbered, from Pirro Nord; IQW 1982/18 052 Mei. 17572 from Untermassfeld^53^; RV41001 from Zhoukoudian Loc. 13^54^; Cey-2-2621 from Ceyssaguet^55^). The measurement protocol follows von den Driesch^56^, with minor modifications, (taken to the nearest 0.1 mm with a digital caliper on physical specimens), whereas for 3D models we used the “measure” function within the software Artec Studio 15 Professional. Morphometric variables include, in alphabetical order: AB, height of the cranium without the sagittal crest (inion-basion); BL, basal length of the cranium (basion-prosthion); Ect, width across the zygomatic processes of the frontals; ECW, width of the muzzle at level of the upper canine; Eu, greatest neurocranium width (eurion-eurion); FL, facial length; GNL, greatest length of the nasals; GPW, greatest palatal width; NCL, neurocranial length; P1-M2 L, length of the upper toothrow, between the mesial side of the P1 and the distal one of the M2; P1-P4 L, length of the premolar row, between the mesial side of the P1 and the distal one of the P4; M1-M2 L, length of the upper molar row, between the mesial side of the M1 and the distal one of the M2; PoCW, smallest width of postorbital constriction; SCL, splanchnocranial length (nasion-prosthion); SH, skull height (with sagittal crest); TL, total length of the cranium (inion-prosthion). See Supplementary Information for visual scheme of measurements used here. Those were selected considering the state of preservation of the fossil specimens (especially *C. arnensis* specimens), in order to maximize their number in the analyses. To these variables, we applied principal component analyses (PCA). The complete dataset used in the PCA is reported in at the following link: 10.5281/zenodo.10418654. The PCA were calculated using the function *prcomp()* (‘*stats*’ package v.4.2.2^57^ and then visualized using *ggplot()* (‘*ggplot2*’ package v.3.4.0^58^).

### Supplementary Information

Supplementary Tables.
